# Health-Related Quality of Life in Chronic Liver Diseases: A Strong Impact of Hand Grip Strength

**DOI:** 10.3390/jcm7120553

**Published:** 2018-12-15

**Authors:** Hiroki Nishikawa, Hirayuki Enomoto, Kazunori Yoh, Yoshinori Iwata, Yoshiyuki Sakai, Kyohei Kishino, Naoto Ikeda, Tomoyuki Takashima, Nobuhiro Aizawa, Ryo Takata, Kunihiro Hasegawa, Noriko Ishii, Yukihisa Yuri, Takashi Nishimura, Hiroko Iijima, Shuhei Nishiguchi

**Affiliations:** Division of Hepatobiliary and Pancreatic disease, Department of Internal Medicine, Hyogo College of Medicine, Hyogo, Nishinomiya 663-8501, Japan; nishikawa_6392_0207@yahoo.co.jp (H.N.); mm2wintwin@ybb.ne.jp (K.Y.); yo-iwata@hyo-med.ac.jp (Y.I.); sakai429@hyo-med.ac.jp (Y.S.); hcm.kyohei@gmail.com (K.K.); nikeneko@hyo-med.ac.jp (N.I.); tomo0204@yahoo.co.jp (T.T.); nobu23hiro@yahoo.co.jp (N.A.); chano_chano_rt@yahoo.co.jp (R.T.); hiro.red1230@gmail.com (K.H.); ishinori1985@yahoo.co.jp (N.I.); gyma27ijo04td@gmail.com (Y.Y.); tk-nishimura@hyo-med.ac.jp (T.N.); hiroko-i@hyo-med.ac.jp (H.I.); nishiguc@hyo-med.ac.jp (S.N.)

**Keywords:** Chronic liver disease, Health-related quality of life, SF-36, Hand grip strength, Skeletal muscle mass

## Abstract

We sought to examine the influence of hand grip strength (HGS) and skeletal muscle mass (SMM) on the health-related quality of life (H-QOL) as evaluated by the 36-Item Short-Form Health Survey (SF-36) questionnaire in chronic liver diseases (CLDs, 198 men and 191 women). Decreased HGS was defined as HGS <26 kg for men and <18 kg for women. Decreased SMM was defined as SMM index <7.0 kg/m^2^ for men and <5.7 kg/m^2^ for women, using bioimpedance analysis. SF-36 scores were compared between groups stratified by HGS or SMM. Between-group differences (decreased HGS vs. non-decreased HGS) in the items of physical functioning (PF), role physical (RP), bodily pain, vitality (VT), social functioning (SF), role emotional (RE), and physical component summary score (PCS) reached significance, while between-group differences (decreased SMM vs. non-decreased SMM) in the items of PF, SF and RE were significant. Multivariate analyses revealed that HGS was significantly linked to PF (*p* = 0.0031), RP (*p* = 0.0185), and PCS (*p* = 0.0421) in males, and PF (*p* = 0.0034), VT (*p* = 0.0150), RE (*p* = 0.0422), and PCS (*p* = 0.0191) in females. HGS had a strong influence especially in the physiological domains in SF-36 in CLDs.

## 1. Introduction

The health-related quality of life (H-QOL) is a patient-centered clinical outcome that is used internationally for patients with various diseases, including diabetes mellitus, and cardiovascular diseases, renal diseases and malignancies [1,2,3,4]. Chronic liver diseases (CLDs) can also affect H-QOL [5,6,7,8,9]. Due to the adverse clinical and patient-reported outcomes, and the economic burden of CLDs, improving H-QOL in patients with CLDs should be a major treatment goal [5,6,7,8,9]. A previous study reported that higher H-QOL was associated with favorable clinical outcome in patients with CLDs and increasing the number of pivotal clinical trials have adopted H-QOL as additional study endpoints [10,11]. The most extensively used assessment methods for H-QOL is the 36-Item Short Form Health Survey (SF-36) [12,13,14,15,16].

Skeletal muscle mass (SMM) increases till 20 years, peaks between 20 and 50 years, and decreases by approximately 1% after the age of 50 years, due to changes in muscle fiber type and size [17]. Sarcopenia is a clinical disease entity that is defined by diminished muscle function and SMM [18,19,20,21,22,23,24,25,26,27,28]. It can be associated with worse clinical outcomes and higher health care costs in various diseases, and there is therefore urgent need for the establishment of methods for improving sarcopenia [18,19,20,21,22,23,24,25,26,27,28]. This also applies to CLD patients [19,21,23]. Sarcopenia is the main component of malnutrition, and it is primarily responsible for the unfavorable clinical consequences that are observed in CLD patients [19,21,23]. Liver cirrhosis (LC) can easily complicate sarcopenia, due to impaired protein synthesis [19]. Currently, the Japanese Society of Hepatology (JSH) reported that the original criteria for sarcopenia in liver diseases, with reference to the Asian criteria for sarcopenia [18,22]. The JSH criteria use hand grip strength (HGS) for muscle strength assessment, and bioimpedance analysis (BIA) and/or computed tomography for muscle mass assessment, while unlike the Asian criteria for sarcopenia, there is no age restriction for the assessment of sarcopenia in the JSH criteria, because younger patients with severe advanced CLDs such as liver failure are likely to be involved in sarcopenia [18].

However, as far as we are aware, scarce data regarding the relevance between H-QOL and HGS and SMM in CLD patients are currently available, although there are several reports where it is not diminished SMM, but diminished muscle strength, that is related to the weakness of physical function in patients with diabetes-related dementia, and that HGS is an important correlate of health in breast cancer survivors [29,30]. We hypothesized that HGS rather than SMM may affect H-QOL in CLD patients. To clarify these clinical research questions, we primarily sought to examine the influence of HGS and SMM on H-QOL, as evaluated by the SF-36 questionnaire, as compared with other clinical data (liver functional data, nutritional data, etc.) in patients with CLDs.

## 2. Patients and Methods

### 2.1. Patients

A total of 410 CLD patients with data for HGS and SMM, using BIA and the SF-36 scores available were admitted to our hospital between November 2013 and August 2018. Each attending physician asked study subjects to receive body composition analysis, and to complete SF-36 questionnaires. Of these, 15 patients with hepatic encephalopathy, advanced malignancies, severe inflammatory diseases, or severe psychiatric diseases that potentially affect H-QOL were excluded. Six subjects with severe ascites were also excluded from the study subjects, because BIA can be challenging in patients with severe fluid retention, that is, overestimates could occur in the calculation of skeletal muscle mass index (SMI) by using BIA in such patients [19]. Three hundred and eighty-nine patients were therefore analyzed. LC was determined using histological and/or imaging studies. FIB-4 index as determined by age, aspartate aminotransferase, alanine aminotransferase, and platelet count, and the controlling nutritional (CONUT) score as determined by serum albumin value, total cholesterol value, and lymphocyte count, were calculated as described elsewhere [31,32].

### 2.2. Questionnaire

Study subjects were asked to complete the Japanese version of the SF-36 (self-reported questionnaire). It consists of 36 items and is classified into multi-item (eight items) scales: physical functioning (PF), role physical (RP), bodily pain (BP), general health perception (GH), vitality (VT), social functioning (SF), role emotion (RE), and mental health (MH) [33]. The physical component summary score (PCS) and the mental component summary score (MCS) were additionally included in this questionnaire, and its validity was well confirmed [33]. Thus, a total of 10 items were evaluated.

### 2.3. Measurement of HGS and SMI

The measurement of HGS was based on the current guidelines [18]. Two measurements of HGS were performed on both the left and right sides. The better measurement on each side was used, and HGS was calculated as the mean of these values. SMI was defined as “appendicular SMM/(height (m))^2^” using BIA. Based on the current guidelines, patients with decreased HGS (d-HGS) were defined as those with HGS < 26 kg for men and <18 kg for women. Similarly, patients with decreased SMM (d-SMM) were defined as those with SMI < 7.0 kg/m^2^ for men and <5.7 kg/m^2^ for women [18]. As described earlier, the JSH criteria for sarcopenia in liver disease determines sarcopenia based on muscle strength and muscle mass regardless of age, because younger patients with advanced LC status may be involved in sarcopenia [18].

First, the impacts of HGS and SMM on the SF-36 scores across 10 items were examined for all cases and several subgroups according to the LC status, gender, and age. Subsequently, the relationships between the SF-36 scores across 10 items and baseline data were investigated. Factors associated with the SF-36 scores were also studied by using multivariate analysis. We received the ethical approval from the ethics committee of our hospital (approval no, 2296). The protocol in the study strictly observed all regulations of the Declaration of Helsinki.

### 2.4. Statistical Considerations

As for continuous parameters, Student’s *t* test, Mann–Whitney *U* test or the Pearson correlation coefficient *r* were employed to assess between-group differences, as applicable. In categorical parameters, Fisher’s exact tests or the Pearson *χ*^2^ test was employed to assess between-group differences, as applicable. Factors with *p* < 0.1 for the correlation with the SF-36 scores across 10 items were subjected to multivariate regression analysis with multiple predictive variables by using the least squares method, to identify candidate parameters. Unless otherwise mentioned, data are indicated as number or average ± standard deviation (SD). All items of SF-36 were analyzed separately, and we considered variables of *p* < 0.05 as being statistically significant variables. The JMP 13.2 (SAS Institute Inc., Cary, NC, USA) was employed to carry out the statistical analysis.

## 3. Results

### 3.1. Patient Baseline Data

Baseline data in this study (*n* = 389, 198 men and 191 women, average age = 62.0 years) are indicated in Table 1. LC was found in 148 patients (38.0%). The average ± SD HGS and SMI in male patients were 33.6 ± 8.7 kg and 7.5 ± 1.1 kg/m^2^, respectively, and those in female patients were 20.9 ± 5.2 kg and 6.0 ± 0.7 kg/m^2^, respectively. Sarcopenia, as defined by the JSH criteria, was observed in 27 male patients (13.6%) and 34 female patients (17.8%) [18]. In LC patients, sarcopenia was identified in 39 patients (26.4%), while in non-LC patients, it was identified in 22 patients (9.1%).

Between-group differences (the d-HGS group (*n* = 93) vs. the non-decreased HGS (nd-HGS) group (*n* = 296)) were noted with statistical significance in age, gender, presence of LC, serum albumin, platelet count, lymphocyte count, total cholesterol, CONUT score, FIB-4 index, and SMI (both male and female). While in the d-SMM group (*n* = 159) vs. the non-decreased SMI (nd-SMM) group (*n* = 230), between-group differences in age, gender, body mass index (BMI), FIB-4 index, serum creatinine, and HGS (both male and female) reached significance. The corresponding average ± SD values and *p* values are listed in Table 1.

Between-group differences (LC patients (*n* = 148) vs. non-LC patients (*n* = 151)) were noted with statistical significance in the items of PF, RP, GH, VT, SF, RE, and PCS, suggesting that LC patients had poorer H-QOL compared with non-LC patients. Corresponding average ± SD values, 95% confidence intervals (CIs) and *P* values were summarized in Table 2.

### 3.2. Impact of HGS and SMM on the SF-36 Scores for all Cases

**Figure 1:**
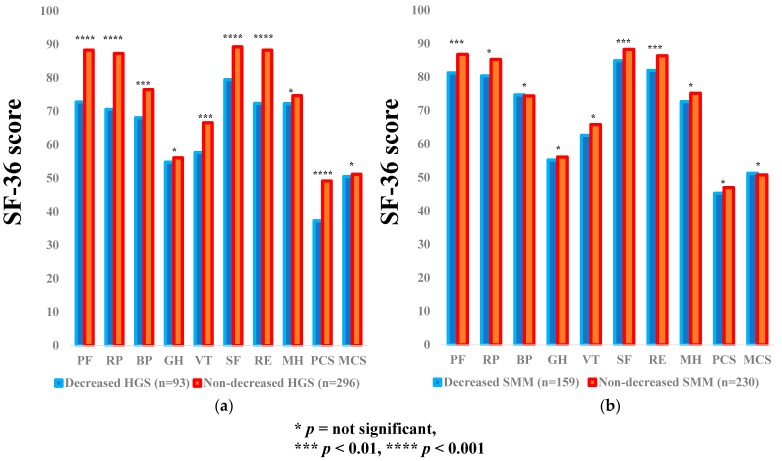
The SF-36 scores across 10 items in the decreased and non-decreased HGS groups (**a**), and the decreased and non-decreased SMM groups (**b**) for all cases (*n* = 389). The average scores in each item of the SF-36 were plotted.

The average ± SD SF-36 scores (95% CIs) in the d-HGS and nd-HGS groups (*n* = 93 and 296), and the d-SMM and nd-SMM groups (*n* = 159 and 230) for all cases are presented in Table 3. Between-group differences (the d-HGS group vs. the nd-HGS group) in the items of PF (*p* < 0.0001), RP (*p* < 0.0001), BP (*p* = 0.0043), VT (*p* = 0.0011), SF (*p* < 0.0001), RE (*p* < 0.0001), and PCS (*p* < 0.0001) reached significance, while SF-36 scores in the d-SMI group were significantly higher than those in the nd-SMI group in the items of PF (*p* = 0.0032), SF (*p* = 0.0031) and RE (*p* = 0.0030). (Figure 1a,b).

### 3.3. Subgroup Analysis 1: Impact of HGS and SMM on the SF-36 Scores for LC Patients

**Figure 2:**
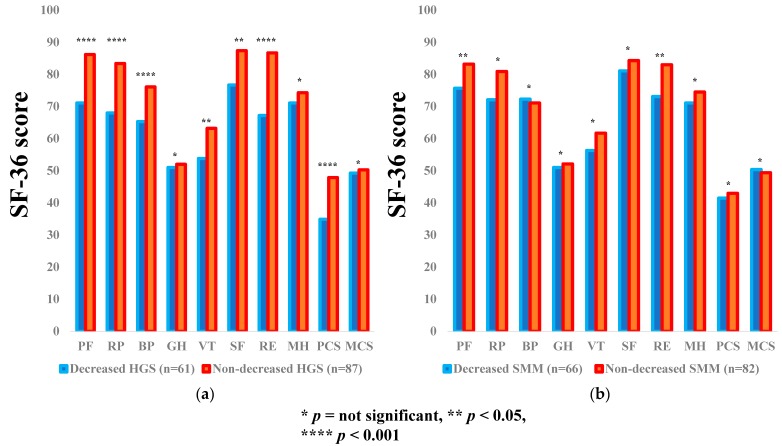
The SF-36 scores across 10 items in the decreased and non-decreased HGS groups (**a**), and the decreased and non-decreased SMM groups (**b**) in LC patients (*n* = 148). The average scores in each item of the SF-36 were plotted.

The average ± SD SF-36 scores [95% CIs] in the d-HGS and nd-HGS groups (*n* = 61 and 87), and the d-SMM and nd-SMM groups (*n* = 66 and 82) for LC patients are shown in Table 3. Between-group differences (the d-HGS group vs. the nd-HGS group) in the items of PF (*p* = 0.0002), RP (*p* = 0.0006), BP (*p* = 0.0002), VT (*p* = 0.0132), SF (*p* = 0.0112), RE (*p* < 0.0001), and PCS (*p* < 0.0001) reached significance, while in the d-SMM group vs. the nd-SMI group, the differences were noted with significance in the items of PF (*p* = 0.0252) and RE (*p* = 0.0131) (Figure 2a,b).

### 3.4. Subgroup Analysis 2: Impact of HGS and SMM on the SF-36 Scores for Non-LC Patients

**Figure 3:**
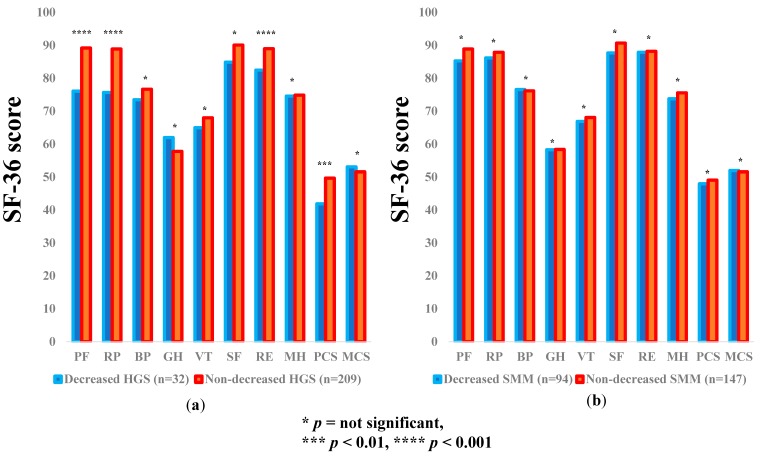
The SF-36 scores across 10 items in the decreased and non-decreased HGS groups (**a**), and the decreased and non-decreased SMM groups (**b**) in non-LC patients (*n* = 241). The average scores in each item of the SF-36 were plotted.

The average ± SD SF-36 scores (95% CIs) in the d-HGS and nd-HGS groups (*n* = 32 and 209) and the d-SMM and nd-SMM groups (*n* = 94 and 147) for non-LC patients are demonstrated in Table 3. Between-group differences (the d-HGS group vs. the nd-HGS group) in the items of PF (*p* = 0.0002), RP (*p* = 0.0006), RE (*p* = 0.0002), and PCS (*p* = 0.0018) reached significance, while in the d-SMM group vs. the nd-SMM group, no significant difference was noted in all items (Figure 3a,b).

### 3.5. Subgroup Analysis 3: Impact of HGS and SMM on the SF-36 Scores for Male Patients

**Figure 4:**
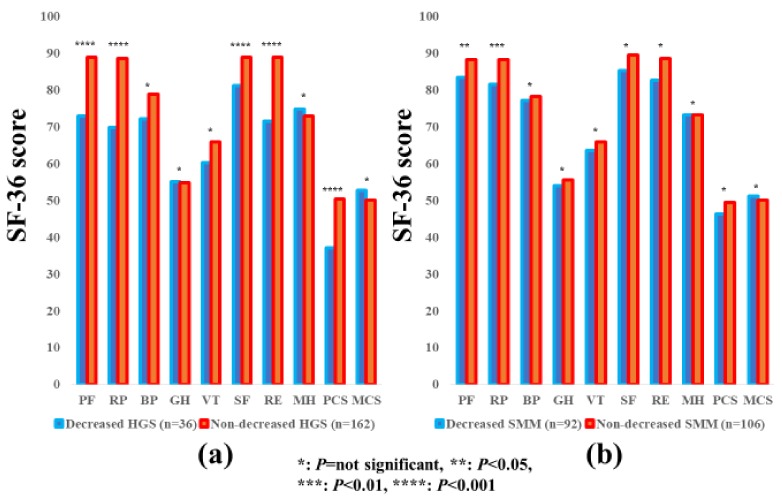
The SF-36 scores across 10 items in the decreased and non-decreased HGS groups (**a**), and the decreased and non-decreased SMM groups (**b**) in male patients (*n* = 198). The average scores in each item of the SF-36 were plotted.

The average ± SD SF-36 scores (95% CIs) in the d-HGS and nd-HGS groups (*n* = 36 and 162) and the d-SMM and nd-SMM groups (*n* = 92 and 106) for male patients are indicated in Table 3. Between-group differences (the d-HGS group vs. the nd-HGS group) in the items of PF (*p* < 0.0001), RP (*p* < 0.0001), SF (*p* < 0.0001), RE (*p* < 0.0001) and PCS (*p* < 0.0001) reached significance, while the SF-36 scores in the d-SMM group were significantly higher than those in the nd-SMM group in the items of PF (*p* = 0.0451) and RP (*p* = 0.0035) (Figure 4a,b).

### 3.6. Subgroup Analysis 4: Impact of HGS and SMM on the SF-36 Scores for Female Patients

**Figure 5:**
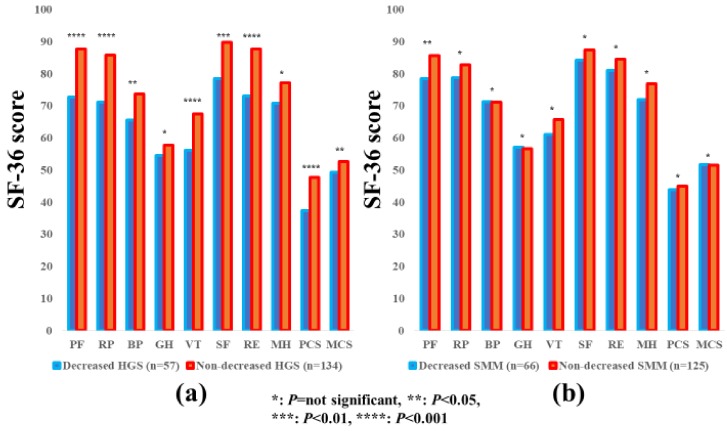
The SF-36 scores across 10 items in the decreased and non-decreased HGS groups (**a**), and the decreased and non-decreased SMM groups (**b**) in female patients (*n* = 191). The average scores in each item of the SF-36 were plotted.

The average ± SD SF-36 scores [95% CIs] in the d-HGS and nd-HGS groups (*n* = 57 and 134) and the d-SMM and nd-SMM groups (*n* = 66 and 125) for female patients are indicated in Table 3. Between-group differences (the d-HGS group vs. the nd-HGS group) in the items of PF (*p* < 0.0001), RP (*p* < 0.0001), BP (*p* = 0.0432), VT (*p* = 0.0006), SF (*p* = 0.0012), RE (*p* < 0.0001), PCS (*p* < 0.0001) and MCS (*p* = 0.0326) reached significance, while in the d-SMM group vs. the nd-SMI group, the difference was observed with significance only in the item of PF (*p* = 0.0124). (Figure 5a,b).

### 3.7. Subgroup Analysis 5: Impact of HGS and SMM on the SF-36 Scores for Patients Aged ≥65 Years

**Figure 6:**
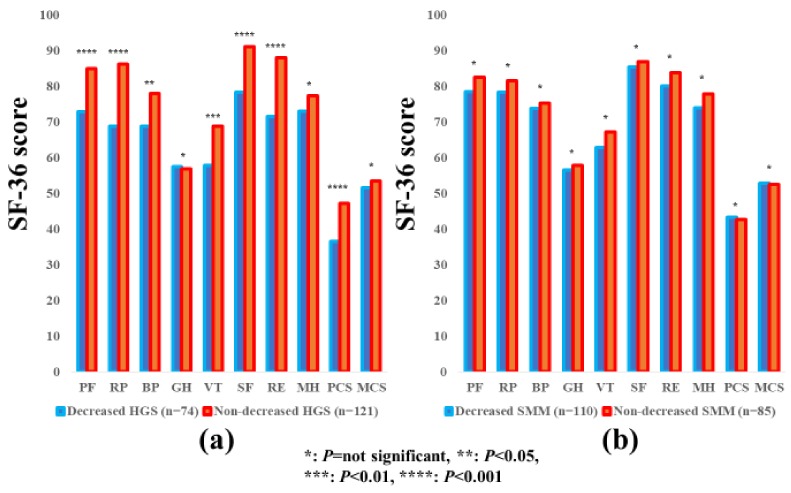
The SF-36 scores across 10 items in the decreased and non-decreased HGS groups (**a**), and the decreased and non-decreased SMM groups (**b**) in patients aged >65 years (*n* = 195). The average scores in each item of the SF-36 were plotted.

The average ± SD SF-36 scores (95% CIs) in the d-HGS and nd-HGS groups (*n* = 74 and 121) and the d-SMM and nd-SMM groups (*n* = 110 and 85) for patients aged ≥65 years are indicated in Table 3. Between-group differences (the d-HGS group vs. the nd-HGS group) in the items of PF (*p* = 0.0001), RP (*p* < 0.0001), BP (*p* = 0.0111), VT (*p* = 0.0021), SF (*p* < 0.0001), RE (*p* < 0.0001), and PCS (*p* < 0.0001) reached significance, while in the d-SMM group vs. the nd-SMM group, no significant differences were observed in all items (Figure 6a,b).

### 3.8. Subgroup Analysis 6: Impact of HGS and SMM on the SF-36 Scores for Patients Aged <65 Years

**Figure 7:**
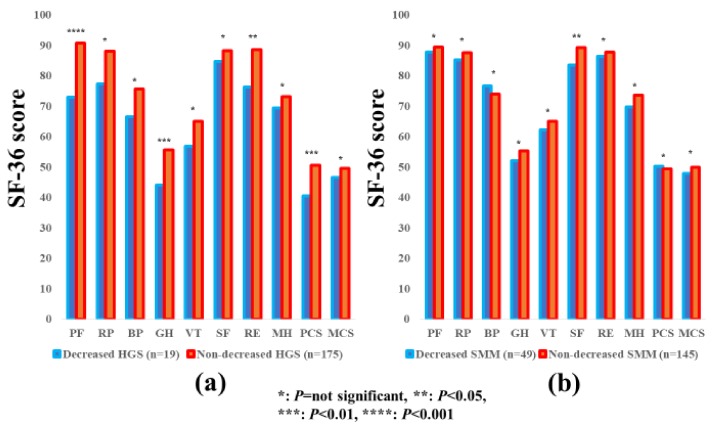
The SF-36 scores across 10 items in the decreased and non-decreased HGS groups (**a**), and the decreased and non-decreased SMM groups (**b**) in patients aged <65 years (*n* = 194). The average scores in each item of the SF-36 were plotted.

The average ± SD SF-36 scores (95% CIs) in the d-HGS and nd-HGS groups (*n* = 19 and 175) and the d-SMM and nd-SMM groups (*n* = 49 and 145) in patients aged <65 years are indicated in Table 3. Between-group differences (the d-HGS group vs. the nd-HGS group) in the items of PF (*p* < 0.0001), GH (*p* = 0.0088), RE (*p* = 0.0281), and PCS (*p* = 0.0081) reached significance, while in the d-SMM group vs. the nd-SMM group, between-group difference was significant only in the item of SF (*p* = 0.0286) (Figure 7a,b).

### 3.9. Relationship between the SF-36 Scores and Baseline Parameters in Male Patients and Female Patients

Correlation coefficients and *p* values between the SF-36 scores across 10 items, and baseline parameters in male patients and female patients are summarized in Table 4.

In male patients, HGS significantly correlated with PF (*r* = 0.32, *p* < 0.0001), RP (*r* = 0.25, *p* = 0.0007), RE (*r* = 0.16, *p* = 0.0324), and PCS (*r* = 0.29, *p* < 0.0001), while SMI did not significantly correlate with any item. Serum albumin level significantly correlated with all items other than MH and HCS.

In female patients, HGS significantly correlated with PF (*r* = 0.35, *p* < 0.0001), RP (*r* = 0.18, *p* = 0.0195), VT (*r* = 0.23, *p* = 0.0022), SF (*r* = 0.18, *p* = 0.0179), RE (*r* = 0.19, *p* = 0.0112), MH (*r* = 0.17, *p* = 0.0222), PCS (*r* = 0.28, *p* = 0.0003), and MCS (*r* = 0.15, *p* = 0.0446), whereas SMI significantly correlated with PF only (*r* = 0.20, *p* = 0.0053). Serum albumin levels significantly correlated with all items other than MH.

### 3.10. Multivariate Analyses of Factors Linked to the SF-36 Scores in Male Patients and Female Patients

In male patients, multivariate analyses of factors for the SF-36 scores revealed that HGS was significantly linked to PF (*p* = 0.0031), RP (*p* = 0.0185) and PCS (*p* = 0.0421), while serum albumin was a significant factor for PF (*p* = 0.0048), RP (*p* = 0.0004), VT (*p* = 0.0327), SF (*p* = 0.0085), RE (*p* = 0.0002) and PCS (*p* < 0.0001) (Table 5).

In female patients, multivariate analyses of factors for the SF-36 scores revealed that HGS was significantly linked to PF (*p* = 0.0034), VT (*p* = 0.0150), RE (*p* = 0.0422), and PCS (*p* = 0.0191), whereas serum albumin did not significantly correlate with any item (Table 5).

## 4. Discussion

The loss of SMM in CLDs is caused by the progressive withdrawal of anabolism, and an increase in catabolism [34]. The liver is an essential organ that is involved in protein, fat, and carbohydrate metabolism, and energy generation [35,36]. H-QOL in CLDs has been demonstrated to be significantly compromised, and the decrease in H-QOL in CLDs is frequently overlooked or unrecognized [37]. To our knowledge, this is the first study demonstrating the relevance between HGS and SMM and H-QOL in CLD patients. To elucidate these issues is clinically of importance, because sarcopenia in liver diseases has been gaining much attention these days, due to its high prognostic predictability, and its definition includes HGS and SMM [18,19,20,21,22,23,24,25,26,27,28]. We therefore conducted the current analysis.

In our results, mean values in the d-HGS and nd-HGS groups were largely different, with statistical significance in the items of PF and PCS for all cases and all subgroup analyses, while in comparison, between the d-SMM and nd-SMM groups, such tendencies were not observed. Additionally, our multivariate analyses revealed that HGS was an independent predictor associated with PF and PCS irrespective of gender; however, in terms of mentality-related domains such as MH or MCS, both HGS and SMI appeared not to have an impact on the SF-36 scores. These results demonstrated that not SMM, but HGS has a strong influence on the physiological domains in the SF-36, which may be linked to clinical outcomes in CLD patients [11]. Numerous previous studies have reported that SMM is an independent outcome predictor in CLD patients [18,19,21,23,25,26]. However, reviewing our current results, decreased SMM itself is not possibly a prognostic factor, but the presence of a large number of patients with diminished muscle strength in diminished SMM patients leads to poor prognosis. Our data showing that SMI was significantly correlated with HGS, both in males (*r* = 0.37, *p* < 0.0001) and females (*r* = 0.46, *p* < 0.0001), support this hypothesis. In this respect, the current results seem to shed some insights on the better understanding of muscle mass and muscle weakness in CLD patients.

Notably, as presented in Table 1, age affected both HGS and SMI, while the presence of LC, serum albumin, and the CONUT score affected only HGS, and BMI affected only SMM. As described above, SMM decreases by approximately 1% after the age of 50 years, owing to changes in muscle fiber type and size [17]. These morphological and functional changes of skeletal muscle due to aging appear to account for the impact of aging on HGS and SMM. On the other hand, our current results denoted that d-SMM does not occur by poor nutritional state alone, and d-HGS does not occur by lower BMI alone, although the mechanisms for these remains unclear. In our comparison of the SF-36 scores across 10 items in patients with and without LC, significant differences were noted in numerous items, which are in agreement with previous reports [19,21,38,39]. LC patients tend to have worse H-QOL and exercise training can be a pivotal recommendation for LC patients [38]. While there have been few reports regarding H-QOL in non-LC patients. In that sense, our data are worthy of report.

In our multivariate analyses, serum albumin was an independent factor in several items of the SF-36 in male patients, while in female patients, serum albumin was not significant in any item of the SF-36. The average ± SD serum albumin levels in male and female in this study were 4.1 ± 0.53 g/dL and 4.2 ± 0.47 g/dL (*p* = 0.4726). Gender differences of hormones, including estrogen and progesterone or physiological and psychological attributes of men and women, may be attributed to our current results, however, it is likely that the ability of protein synthesis is associated with H-QOL in male CLD patients [40,41]. In our previous investigation, we have reported that serum levels of myostatin, which is a negative regulator of muscle protein synthesis, significantly differed in male and female LC patients [23].

The proportions of sarcopenia in LC and non-LC patients were 26.4% and 9.1%, respectively, in this study. Sarcopenia includes primary and secondary sarcopenia [18]. LC patients may have secondary sarcopenia due to impaired protein synthesis, and non-LC patients may have aging-related primary sarcopenia [18]. Clinicians should fully consider the etiology for sarcopenia in each patient.

Several limitations related to the study warrant mention. Firstly, the study was a single-center observational study with a retrospective nature. Secondly, the study data was derived from a Japanese liver disease population data, and additional investigations on other races are required to further verify and extend the application to other races. Thirdly, HGS can vary depending on patients’ daily life activities. Fourthly, patients with massive ascites or hepatic encephalopathy, who are potentially involved in sarcopenia were excluded, due to the lack of reliability in the BIA or the self-reported questionnaire, creating bias. Finally, the interpretation of our results should be done cautiously, since the direction of the association between the SF-36 scores and HGS or SMM remains unclear, due to the cross-sectional nature of our data. Nevertheless, our study results denoted that patients with d-HGS scored lower in the SF-36 vs. those with nd-HGS, especially in the physical health domains. In conclusion, HGS appears to have a strong impact on H-QOL in patients with CLDs, and exercise may be beneficial for improving H-QOL. In CLD patients with d-HGS, clinicians should be aware of the presence of CLD patients with decreased H-QOL.

## 5. Conclusions

HGS appears to have a strong impact on H-QOL in patients with CLDs.

## Figures and Tables

**Table 1:** Baseline characteristics.

Variables	All Cases (*n* = 389)	Decreased HGS (*n* = 93)	Non-Decreased HGS (*n* = 296)	*p* Value	Decreased SMM (*n* = 159)	Non-decreased SMM (*n* = 230)	*p* Value
Age (years)	62.0 ± 12.8	69.7 ± 10.6	59.5 ± 12.5	<0.0001	66.7 ± 12.2	58.7 ± 12.2	<0.0001
Gender, male/female	198/191	36/57	162/134	0.0087	93/66	105/125	0.0135
Etiology, HBV/HCV/HBV, and HCV/NBNC	61/234/8/86	10/60/2/21	51/174/6/65	0.5130	21/101/5/32	40/133/3/54	0.3102
Presence of LC, yes/no	148/241	61/32	87/209	<0.0001	65/94	83/147	0.3416
Body mass index (kg/m^2^)	23.2 ± 3.8	22.7 ± 3.3	23.3 ± 4.0	0.1554	20.9 ± 2.4	24.8 ± 3.8	<0.0001
Total bilirubin (mg/dL)	0.96 ± 0.58	0.96 ± 0.62	0.96 ± 0.57	0.9545	0.86 ± 0.39	1.02 ± 0.68	0.0718
Serum albumin (g/dL)	4.1 ± 0.5	3.9 ± 0.57	4.2 ± 0.45	<0.0001	4.2 ± 0.48	4.1 ± 0.52	0.2688
Prothrombin time (%)	86.7 ± 16.6	84.1 ± 17.1	87.5 ± 16.3	0.0790	87.5 ± 16.9	86.1 ± 16.3	0.4112
Platelet count (×10^4^/mm^3^)	16.8 ± 6.9	15.1 ± 6.5	17.3 ± 6.9	0.0081	16.7 ± 6.2	16.8 ± 7.3	0.7315
White blood cell (/mm^3^)	5165 ± 1614	5193 ± 1659	5156 ± 1602	0.8455	5240 ± 1466	5113 ± 1710	0.3114
Lymphocyte count (/mm^3^)	3112 ± 958	1395 ± 598	1627 ± 613	0.0015	1513 ± 542	1613 ± 661	0.2045
Total cholesterol (mg/dL)	181.3 ± 41.3	170.5 ± 47.2	184.7 ± 38.8	0.0038	182.0 ± 43.9	180.8 ± 39.5	0.7775
CONUT score	1.9 ± 1.9	2.7 ± 2.5	1.6 ± 1.6	0.0003	1.9 ± 1.7	1.9 ± 2.0	0.8450
AST (IU/L)	37.7 ± 27.3	40.3 ± 29.2	36.8 ± 26.6	0.2886	36.9 ± 26.1	38.2 ± 28.1	0.6286
ALT (IU/L)	37.3 ± 37.3	35.0 ± 38.4	38.1 ± 37.0	0.4966	35.4 ± 36.3	38.7 ± 38.0	0.3828
FIB-4 index	3.3 ± 3.3	4.0 ± 2.8	3.1 ± 3.4	0.0142	3.2 ± 2.2	3.4 ± 3.9	0.0215
Serum creatinine (mg/dL)	0.73 ± 0.54	0.76 ± 0.53	0.72 ± 0.55	0.4769	0.81 ± 0.81	0.67 ± 0.18	0.0095
HbA1c (NGSP)	5.9 ± 0.88	5.9 ± 0.80	5.8 ± 0.90	0.7085	5.9 ± 0.84	5.8 ± 0.90	0.7085
Serum sodium (mmol/L)	140.1 ± 2.3	139.9 ± 2.5	140.2 ± 2.3	0.2330	140.3 ± 2.4	140.0 ± 2.3	0.3413
HGS (kg), male	33.6 ± 8.7	22.0 ± 3.9	36.3 ± 6.4	<0.0001	29.4 ± 6.9	37.4 ± 7.5	<0.0001
HGS (kg), female	20.9 ± 5.2	14.8 ± 3.1	23.3 ± 3.6	<0.0001	18.2 ± 4.1	22.4 ± 5.1	<0.0001
SMI (kg/m^2^), male	7.5 ± 1.1	7.0 ± 0.97	7.6 ± 1.09	0.0039	6.8 ± 0.43	8.1 ± 1.13	<0.0001
SMI (kg/m^2^), female	6.0 ± 0.7	5.6 ± 0.74	6.1 ± 0.68	<0.0001	5.2 ± 0.39	6.4 ± 0.50	<0.0001

Data are expressed as average ± standard deviation. HBV; hepatitis B virus, HCV; hepatitis C virus, NBNC; non-B and non-C, LC; liver cirrhosis, CONUT score; controlling nutritional score, AST; aspartate aminotransferase, ALT; alanine aminotransferase, NGSP; National Glycohemoglobin Standardization Program, HGS; hand grip strength, SMM; skeletal muscle mass, SMI; skeletal muscle mass index.

**Table 2:** Comparison of the SF-36 scores across 10 items in patients with LC and non-LC.

ALL Cases	LC Patients (*n* = 148)	Non-LC Patients (*n* = 241)	*p* Value
PF	79.9 ± 20.2 [76.6, 83.2]	87.5 ± 15.8 [85.5, 89.5]	<0.0001
RP	77.0 ± 30.7 [72.0, 82.0]	87.2 ± 21.4 [84.5, 90.0]	0.0005
BP	71.6 ± 26.1 [67.3, 75.9]	76.3 ± 23.9 [73.3, 79.3]	0.0709
GH	51.6 ± 19.7 [48.4, 54.8]	58.4 ± 19.1 [55.9, 60.9]	0.0011
VT	59.3 ± 22.6 [55.6, 63.0]	67.6 ± 20.5 [65.0, 70.2]	0.0002
SF	82.9 ± 25.1 [78.8, 87.0]	89.4 ± 19.4 [86.9, 91.9]	0.0004
RE	78.6 ± 28.3 [74.0, 83.3]	88.1 ± 21.4 [85.4, 90.8]	<0.0001
MH	73.0 ± 20.6 [69.6, 76.4]	74.9 ± 20.7 [72.2, 77.5]	0.3845
PCS	42.3 ± 16.2 [39.6, 45.1]	48.7 ± 11.9 [47.1, 50.2]	<0.0001
MCS	49.9 ± 10.1 [48.2, 51.6]	51.8 ± 10.4 [50.4, 53.1]	0.0891

Data are expressed as average ± standard deviation and 95% confidence interval. LC; liver cirrhosis, PF; physical functioning, RP; role physical, BP; bodily pain, GH; general health perception, VT; vitality, SF; social functioning, RE; role emotion, MH; mental health, PCS; physical component summary score, MCS; mental component summary score.

**Table 3:** Comparison of the SF-36 scores across 10 items.

**ALL Cases**	**Decreased HGS (*n* = 93)**	**Non-Decreased HGS (*n* = 296)**	***p* Value**
PF	72.8 ± 22.6 [68.1, 77.5]	88.3 ± 14.3 [86.7, 90.0]	<0.0001
RP	70.6 ± 33.1 [63.7, 77.4]	87.3 ± 21.7 [84.8, 89.8]	<0.0001
BP	68.1 ± 24.0 [63.2, 73.1]	76.5 ± 24.8 [73.7, 79.4]	0.0043
GH	54.8 ± 22.7 [50.1, 59.5]	56.1 ± 18.5 [53.9, 58.2]	0.6190
VT	57.7 ± 25.1 [52.5, 62.9]	66.6 ± 20.0 [64.3, 68.9]	0.0011
SF	79.5 ± 25.4 [74.2, 84.8]	89.3 ± 20.2 [86.9, 91.6]	<0.0001
RE	72.4 ± 30.3 [66.1, 78.8]	88.3 ± 21.2 [85.9, 90.7]	<0.0001
MH	72.3 ± 24.1 [67.3, 77.3]	74.7 ± 19.4 [72.5, 77.0]	0.7439
PCS	37.3 ± 16.7 [33.8, 40.8]	49.2 ± 11.7 [47.8, 50.6]	<0.0001
MCS	50.5 ± 11.0 [48.2, 52.9]	51.2 ± 10.1 [50.5, 52.4]	0.5941
**ALL Cases**	**Decreased SMM (*n* = 159)**	**Non-Decreased SMM (*n* = 230)**	***p* Value**
PF	81.3 ± 18.1 [78.5, 84.2]	86.8 ± 17.5 [84.6, 89.1]	0.0032
RP	80.4 ± 27.2 [76.2, 84.7]	85.3 ± 24.7 [82.1, 88.6]	0.0660
BP	74.7 ± 25.1 [70.8, 78.7]	74.4 ± 24.6 [71.2, 77.6]	0.8891
GH	55.2 ± 20.4 [52.0, 58.5]	56.1 ± 19.1 [53.6, 58.7]	0.6656
VT	62.6 ± 23.6 [58.9, 66.3]	65.8 ± 20.2 [63.2, 68.4]	0.3364
SF	84.9 ± 24.7 [80.9, 88.8]	88.3 ± 19.7 [85.7, 91.0]	0.0031
RE	82.0 ± 26.6 [77.8, 86.1]	86.4 ± 23.0 [83.3, 89.4]	0.0030
MH	72.7 ± 22.4 [69.2, 76.2]	75.2 ± 19.3 [72.7, 77.7]	0.4683
PCS	45.3 ± 14.2 [43.0, 47.6]	47.0 ± 13.9 [45.1, 48.8]	0.2610
MCS	51.3 ± 10.5 [49.6, 53.0]	50.8 ± 10.1 [49.5, 52.2]	0.6601
**LC**	**Decreased HGS (*n* = 61)**	**Non-Decreased HGS (*n* = 87)**	***p* Value**
PF	71.1 ± 23.1 [65.2, 77.1]	86.2 ± 15.0 [82.9, 89.4]	0.0002
RP	68.0 ± 34.0 [59.3, 76.7]	83.4 ± 26.5 [77.7, 89.1]	0.0006
BP	65.3 ± 22.8 [59.5, 71.2]	76.1 ± 27.5 [70.2, 82.0]	0.0002
GH	51.0 ± 19.9 [46.0, 56.1]	52.0 ± 19.7 [47.7, 56.3]	0.7691
VT	53.8 ± 23.7 [47.7, 59.9]	63.2 ± 21.1 [58.7, 67.7]	0.0132
SF	76.7 ± 26.8 [69.8, 83.6]	87.4 ± 22.9 [82.4, 92.3]	0.0112
RE	67.2 ± 30.9 [59.3, 75.2]	86.7 ± 23.3 [81.7, 91.7]	<0.0001
MH	71.1 ± 22.8 [65.2, 77.0]	74.3 ± 18.9 [70.3, 78.4]	0.3514
PCS	34.9 ± 16.5 [30.7, 39.2]	47.9 ± 13.7 [44.8, 50.9]	<0.0001
MCS	49.3 ± 9.7 [46.8, 51.8]	50.3 ± 10.5 [48.0, 52.7]	0.5382
**LC**	**Decreased SMM (*n* = 66)**	**Non-Decreased SMM (*n* = 82)**	***p* Value**
PF	75.7 ± 20.7 [70.5, 80.8]	83.2 ± 19.2 [79.0, 87.4]	0.0252
RP	72.1 ± 31.6 [64.3, 80.0]	80.9 ± 29.6 [74.4, 87.4]	0.0867
BP	72.3 ± 26.1 [65.8, 78.8]	71.1 ± 26.3 [65.3, 76.8]	0.7832
GH	51.0 ± 20.0 [46.1, 56.0]	52.1 ± 19.6 [47.7, 56.4]	0.7589
VT	56.3 ± 24.3 [50.2, 62.4]	61.7 ± 21.0 [57.1, 66.3]	0.1519
SF	81.1 ± 28.4 [74.1, 88.3]	84.3 ± 22.1 [79.4, 89.2]	0.4615
RE	73.1 ± 31.9 [65.2, 81.1]	83.0 ± 24.4 [77.6, 88.4]	0.0131
MH	71.1 ± 23.7 [65.2, 77.0]	74.5 ± 17.8 [70.6, 78.4]	0.6602
PCS	41.5 ± 17.2 [37.2, 45.8]	43.0 ± 15.5 [39.5, 46.6]	0.5729
MCS	50.4 ± 10.4 [47.8, 53.1]	49.4 ± 9.9 [47.2, 51.7]	0.5615
**Non-LC**	**Decreased HGS (*n* = 32)**	**Non-Decreased HGS (*n* = 209)**	***p* Value**
PF	76.1 ± 21.6 [68.1, 84.0]	89.2 ± 14.0 [87.3, 91.1]	<0.0001
RP	75.7 ± 30.9 [64.4, 87.1]	88.9 ± 19.2 [86.3, 91.6]	<0.0001
BP	73.5 ± 25.6 [64.3, 82.7]	76.7 ± 23.6 [73.5, 79.9]	0.4782
GH	62.0 ± 26.2 [52.5, 71.5]	57.8 ± 17.7 [55.3, 60.3]	0.0876
VT	65.0 ± 26.5 [55.5, 74.6]	68.0 ± 19.4 [65.4, 70.7]	0.4422
SF	84.9 ± 22.1 [76.8, 93.0]	90.1 ± 18.9 [87.5, 92.7]	0.1629
RE	82.5 ± 27.0 [72.6, 92.4]	89.0 ± 20.3 [86.2, 91.7]	0.0002
MH	74.6 ± 26.6 [65.0, 84.2]	74.9 ± 19.7 [72.2, 77.6]	0.4130
PCS	41.9 ± 16.4 [35.8, 48.0]	49.7 ± 10.6 [48.2, 51.2]	0.0018
MCS	53.1 ± 13.1 [48.2, 58.0]	51.6 ± 9.9 [40.2, 52.9]	0.3525
**Non-LC**	**Decreased SMM (*n* = 94)**	**Non-Decreased SMM (*n* = 147)**	***p* Value**
PF	85.3 ± 14.9 [82.2, 88.3]	88.9 ± 16.2 [86.3, 91.5]	0.0830
RP	86.2 ± 21.9 [81.7, 90.7]	87.9 ± 21.2 [84.4, 91.3]	0.5689
BP	76.6 ± 24.4 [71.4, 81.5]	76.2 ± 23.6 [72.4, 80.1]	0.9476
GH	58.3 ± 20.2 [54.0, 62.5]	58.4 ± 18.5 [55.4, 61.5]	0.9482
VT	66.9 ± 22.2 [62.4, 71.5]	68.1 ± 19.4 [64.9, 71.2]	0.6748
SF	87.7 ± 21.5 [83.0, 91.9]	90.7 ± 17.8 [87.7, 93.6]	0.2185
RE	87.9 ± 20.5 [83.8, 92.1]	88.2 ± 22.0 [84.6, 91.8]	0.9205
MH	73.8 ± 21.5 [69.3, 78.2]	75.6 ± 20.2 [72.3, 78.9]	0.5102
PCS	48.0 ± 11.1 [45.7, 50.3]	49.1 ± 12.4 [47.1, 51.2]	0.4689
MCS	52.0 ± 10.6 [49.7, 54.2]	51.6 ± 10.2 [49.9, 53.3]	0.8203
**Male**	**Decreased HGS (*n* = 36)**	**Non-Decreased HGS (*n* = 162)**	***p* Value**
PF	72.9 ± 23.7 [64.9, 80.9]	88.9 ± 13.9 [86.7, 91.1]	<0.0001
RP	69.8 ± 34.9 [58.0, 81.6]	88.6 ± 20.1 [85.4, 91.7]	<0.0001
BP	72.2 ± 26.0 [63.4, 81.0]	78.9 ± 23.6 [75.2, 82.5]	0.1328
GH	55.1 ± 23.1 [47.3, 62.9]	54.7 ± 18.3 [51.8, 57.6]	0.9145
VT	60.2 ± 26.3 [51.1, 69.2]	65.8 ± 20.5 [62.7, 69.0]	0.1614
SF	81.2 ± 27.2 [71.8, 90.5]	88.9 ± 20.3 [85.7, 92.1]	<0.0001
RE	71.5 ± 33.2 [60.1, 82.9]	88.9 ± 20.8 [85.6, 92.1]	<0.0001
MH	74.8 ± 24.0 [66.6, 83.1]	72.9 ± 20.0 [69.7, 76.0]	0.6112
PCS	37.0 ± 18.7 [30.6, 43.4]	50.4 ± 11.6 [48.5, 52.3]	<0.0001
MCS	52.7 ± 11.5 [48.8, 56.7]	50.1 ± 10.3 [48.4, 51.7]	0.1817
**Male**	**Decreased SMM (*n* = 92)**	**Non-Decreased SMM (*n* = 106)**	***p* Value**
PF	83.4 ± 16.5 [80.0. 86.8]	88.3 ± 17.5 [84.9, 91.7]	0.0451
RP	81.6 ± 26.8 [76.1, 87.1]	88.3 ± 21.8 [84.1, 92.6]	0.0035
BP	77.1 ± 24.8 [72.0, 82.2]	78.2 ± 23.6 [73.6, 82.7]	0.7508
GH	53.9 ± 20.9 [49.6, 58.3]	55.5 ± 17.8 [52.0, 59.0]	0.5712
VT	63.6 ± 24.5 [58.6, 68.7]	65.9 ± 18.9 [62.2, 69.5]	0.9880
SF	85.3 ± 25.0 [80.1, 90.5]	89.5 ± 18.6 [85.8, 93.1]	0.0507
RE	82.6 ± 27.1 [77.0, 88.2]	88.5 ± 21.3 [84.4, 92.7]	0.0904
MH	73.2 ± 22.5 [68.6, 77.9]	73.2 ± 19.2 [69.5, 76.9]	0.9942
PCS	46.3 ± 14.6 [43.3, 49.4]	49.4 ± 13.7 [46.6, 52.1]	0.1419
MCS	51.1 ± 11.2 [48.7, 53.4]	50.1 ± 10.1 [48.1, 52.1]	0.5428
**Female**	**Decreased HGS (*n* = 57)**	**Non-Decreased HGS (*n* = 134)**	***p* Value**
PF	72.7 ± 22.1 [66.8, 78.7]	87.6 ± 14.9 [85.0, 90.1]	<0.0001
RP	71.1 ± 32.2 [62.5, 79.7]	85.8 ± 23.4 [81.8, 89.8]	<0.0001
BP	65.6 ± 22.5 [59.6, 71.6]	73.6 ± 25.9 [69.2, 78.1]	0.0432
GH	54.6 ± 22.7 [48.6, 60.6]	57.8 ± 18.6 [54.5, 61.0]	0.3266
VT	56.2 ± 24.5 [49.7, 62.7]	67.5 ± 19.4 [64.2, 70.9]	0.0006
SF	78.4 ± 24.5 [71.9, 85.0]	89.7 ± 20.1 [86.2, 93.3]	0.0012
RE	73.0 ± 8.7 [65.3, 80.7]	87.6 ± 21.7 [83.9, 91.3]	<0.0001
MH	70.8 ± 24.3 [64.3, 77.2]	77.1 ± 18.5 [73.9, 80.3]	0.1394
PCS	37.4 ± 15.5 [33.2, 41.6]	47.7 ± 11.6 [45.7, 49.8]	<0.0001
MCS	49.2 ± 10.5 [46.3, 52.0]	52.6 ± 9.6 [50.9, 54.3]	0.0326
**Female**	**Decreased SMM (*n* = 66)**	**Non-Decreased SMM (*n* = 125)**	***p* Value**
PF	78.5 ± 19.9 [73.6, 83.5]	85.6 ± 17.5 [82.5, 88.7]	0.0124
RP	78.8 ± 27.8 [71.9, 85.7]	82.8 ± 26.7 [78.1, 87.6]	0.3273
BP	71.3 ± 25.3 [65.0, 77.6]	71.1 ± 25.2 [66.7, 75.6]	0.9672
GH	57.1 ± 19.7 [52.2, 62.0]	56.6 ± 20.2 [53.0, 60.3]	0.8853
VT	61.1 ± 22.3 [55.6, 66.6]	65.7 ± 21.2 [61.9, 69.5]	0.1652
SF	84.2 ± 24.5 [78.2, 90.3]	87.4 ± 20.6 [83.7, 91.2]	0.3527
RE	81.0 ± 26.2 [74.6, 87.5]	84.5 ± 24.1 [80.3, 88.8]	0.3546
MH	71.9 ± 22.4 [66.4, 77.5]	76.9 ± 19.4 [73.4, 80.3]	0.1161
PCS	43.9 ± 13.7 [40.4, 47.3]	45.0 ± 13.8 [42.5, 47.5]	0.5979
MCS	51.7 ± 9.7 [49.3, 54.1]	51.5 ± 10.2 [49.6, 53.3]	0.8719
**≥65 years**	**Decreased HGS (*n* = 74)**	**Non-Decreased HGS (*n* = 121)**	***p* Value**
PF	72.8 ± 23.5 [67.3, 78.3]	84.9 ± 14.0 [82.3, 87.4]	0.0001
RP	68.8 ± 34.6 [60.8, 76.9]	86.3 ± 20.6 [82.6, 90.1]	<0.0001
BP	68.8 ± 24.3 [63.2, 74.4]	78.0 ± 24.1 [73.6, 82.4]	0.0111
GH	57.6 ± 23.3 [52.2, 63.0]	56.8 ± 18.9 [53.3, 60.3]	0.7950
VT	57.9 ± 25.7 [52.0. 63.9]	68.9 ± 19.8 [65.3, 72.5]	0.0021
SF	78.3 ± 25.1 [72.5, 84.1]	91.1 ± 18.3 [87.7, 94.4]	<0.0001
RE	71.5 ± 30.6 [64.4, 78.7]	88.0 ± 20.5 [84.2, 91.7]	<0.0001
MH	73.0 ± 25.1 [67.2, 78.8]	77.4 ± 17.9 [74.1, 80.6]	0.5509
PCS	36.5 ± 17.6 [32.4, 40.6]	47.2 ± 10.1 [45.3, 49.1]	<0.0001
MCS	51.5 ± 11.2 [48.9, 54.1]	53.5 ± 9.2 [51.8, 55.2]	0.1822
**≥65 years**	**Decreased SMM (*n* = 110)**	**Non-Decreased SMM (*n* = 85)**	***p* Value**
PF	78.5 ± 18.6 [75.0, 82.1]	82.6 ± 19.4 [78.4, 86.8]	0.1435
RP	78.3 ± 28.6 [72.8, 83.7]	81.6 ± 27.4 [75.6, 87.5]	0.4226
BP	73.9 ± 26.2 [68.9, 78.9]	75.2 ± 22.4 [70.3, 80.0]	0.7338
GH	56.6 ± 21.9 [52.4, 60.8]	57.8 ± 19.0 [53.6, 62.0]	0.6926
VT	62.8 ± 24.4 [58.2, 67.4]	67.2 ± 20.6 [62.8, 71.7]	0.1809
SF	85.5 ± 23.8 [80.9, 90.0]	86.9 ± 19.5 [82.6, 91.2]	0.6548
RE	80.1 ± 27.3 [74.9, 85.2]	83.9 ± 24.2 [78.6, 89.3]	0.3090
MH	74.0 ± 22.3 [69.8, 78.2]	77.9 ± 19.1 [73.8, 82.1]	0.1972
PCS	43.3 ± 14.1 [40.6, 45.9]	42.7 ± 15.2 [39.3, 46.1]	0.7951
MCS	52.8 ± 10.1 [50.9, 54.7]	52.6 ± 10.1 [50.4, 54.9]	0.9288
**<65 years**	**Decreased HGS (*n* = 19)**	**Non-Decreased HGS (*n* = 175)**	***p* Value**
PF	72.8 ± 19.6 [63.3, 82.2]	90.7 ± 14.1 [88.6, 92.8]	<0.0001
RP	77.3 ± 25.9 [64.8, 89.8]	88.0 ± 22.4 [84.6, 91.3]	0.0530
BP	65.5 ± 23.1 [54.4, 76.7]	75.5 ± 25.2 [71.8, 79.3]	0.0990
GH	44.0 ± 16.9 [35.8, 52.1]	55.6 ± 18.3 [52.8, 58.4]	0.0088
VT	56.8 ± 23.2 [45.3, 68.3]	65.0 ± 20.0 [62.0, 68.0]	0.1042
SF	84.6 ± 27.1 [70.7, 98.6]	88.1 ± 21.3 [84.9, 91.3]	0.5338
RE	76.2 ± 29.8 [61.4, 91.0]	88.5 ± 21.7 [85.3, 91.8]	0.0281
MH	69.4 ± 20.2 [59.3, 79.4]	73.0 ± 20.3 [70.0, 76.0]	0.4708
PCS	40.5 ± 11.8 [34.4, 46.6]	50.5 ± 12.5 [48.6, 52.4]	0.0018
MCS	46.5 ± 9.4 [41.7, 51.3]	49.6 ± 10.3 [48.1, 51.2]	0.2286
**<65 years**	**Decreased SMM (*n* = 49)**	**Non-Decreased SMM (*n* = 145)**	***p* Value**
PF	87.7 ± 15.1 [83.4, 92.1]	89.3 ± 15.8 [86.7, 91.9]	0.5386
RP	85.2 ± 23.3 [78.6, 91.9]	87.5 ± 22.8 [83.8, 91.3]	0.5501
BP	76.5 ± 22.8 [69.9, 83.0]	73.9 ± 25.9 [69.7, 78.2]	0.5389
GH	52.0 ± 16.1 [47.3, 56.8]	55.2 ± 19.1 [52.0, 58.4]	0.3182
VT	62.2 ± 21.9 [55.8, 68.5]	65.0 ± 19.9 [61.7, 68.2]	0.4133
SF	83.4 ± 26.9 [75.5, 91.4]	89.2 ± 19.8 [85.9, 92.5]	0.0286
RE	86.3 ± 24.8 [79.1, 93.5]	87.7 ± 22.2 [84.1, 91.4]	0.7097
MH	69.7 ± 22.5 [63.2, 76.3]	73.6 ± 19.4 [70.4, 76.8]	0.2510
PCS	50.2 ± 13.6 [46.1, 54.3]	49.4 ± 12.5 [47.3, 51.5]	0.7120
MCS	47.9 ± 10.9 [44.6, 51.1]	49.8 ± 10.0 [48.2, 51.5]	0.2593

Data are expressed as average ± standard deviation and 95% confidence interval. HGS; hand grip strength, SMM; skeletal muscle mass, LC; liver cirrhosis, PF; physical functioning, RP; role physical, BP; bodily pain, GH; general health perception, VT; vitality, SF; social functioning, RE; role emotion, MH; mental health, PCS; physical component summary score, MCS; mental component summary score.

**Table 4:** Relationship between baseline data and the SF-36 scores in each item in male and female patients.

**Male**	**PF**		**RP**		**BP**		**GH**		**VT**	
	***r***	***p* Value**	***r***	***p* Value**	***r***	***p* Value**	***r***	***p* Value**	***r***	***p* Value**
Age	−0.21	0.0036	−0.12	0.0939	−0.12	0.0869	0.11	0.1298	0.069	0.3346
BMI	−0.057	0.4246	−0.051	0.4740	−0.047	0.5091	0.039	0.5925	−0.044	0.5394
HGS	0.32	<0.0001	0.25	0.0007	0.048	0.5242	−0.016	0.8924	0.0058	0.9383
SMI	−0.0066	0.9268	−0.0028	0.9685	−0.081	0.2577	−0.00035	0.9961	−0.062	0.3920
Albumin	0.42	<0.0001	0.38	<0.0001	0.21	0.0025	0.18	0.0143	0.25	0.0005
Bilirubin	0.011	0.8784	−0.021	0.7731	−0.041	0.5686	−0.061	0.4026	−0.0278	0.7018
PT	0.26	0.0002	0.16	0.0245	0.063	0.3773	0.17	0.0209	0.19	0.0086
Platelet	0.11	0.1250	0.042	0.5583	−0.031	0.6653	0.11	0.1449	0.020	0.7802
CONUT	−0.4	<0.0001	−0.30	<0.0001	−0.12	0.0924	−0.17	0.0164	−0.17	0.0181
AST	−0.01	0.1626	−0.27	0.0013	−0.15	0.0412	−0.21	0.0030	−0.16	0.0220
ALT	−0.027	0.7035	−0.18	0.0104	−0.11	0.1393	−0.17	0.0189	−0.13	0.0707
**Male**	**SF**		**RE**		**MH**		**PCS**		**MCS**	
	***r***	***p* Value**	***r***	***p* Value**	***r***	***p* Value**	***r***	***p* Value**	***r***	***p* Value**
Age	−0.019	0.7916	−0.064	0.3732	0.14	0.0509	−0.24	0.0011	0.22	0.0030
BMI	−0.046	0.5294	0.055	0.4441	0.031	0.6637	−0.083	0.260	0.0099	0.8930
HGS	0.10	0.1827	0.16	0.0324	−0.10	0.1772	0.29	0.0001	−0.16	0.0353
SMI	−0.043	0.5601	0.056	0.4391	0.046	0.5193	−0.050	0.4976	−0.02	0.7834
Albumin	0.27	0.0002	0.33	<0.0001	0.086	0.2273	0.46	<0.0001	0.046	0.5348
Bilirubin	−0.064	0.3845	−0.0071	0.9215	0.067	0.3569	−0.02	0.7925	−0.035	0.6371
PT	0.16	0.0286	0.10	0.1503	0.020	0.7849	0.19	0.0088	0.069	0.3468
Platelet	0.10	0.1614	−0.040	0.5762	−0.1243	0.0818	0.051	0.4905	−0.023	0.7590
CONUT	−0.19	0.0091	−0.21	0.0031	0.026	0.7197	0.36	<0.0001	0.011	0.8773
AST	−0.070	0.3345	−0.19	0.0071	−0.13	0.0675	−0.16	0.0286	−0.12	0.1021
ALT	−0.025	0.7311	−0.20	0.0050	−0.15	0.0331	0.10	0.1650	−0.11	0.1172
**Female**	**PF**		**RP**		**BP**		**GH**		**VT**	
	***r***	***p* Value**	***r***	***p* Value**	***r***	***p* Value**	***r***	***p* Value**	***r***	***p* Value**
Age	−0.25	0.0004	−0.15	0.0384	0.051	0.4835	0.077	0.2987	−0.035	0.6346
BMI	−0.037	0.6128	−0.0065	0.9292	−0.025	0.7329	−0.0248	0.7383	0.0059	0.9357
HGS	0.35	<0.0001	0.18	0.0195	0.098	0.1953	0.054	0.4815	0.23	0.0022
SMI	0.20	0.0053	0.10	0.1677	−0.048	0.5136	−0.0055	0.9409	0.096	0.1877
Albumin	0.24	0.0011	0.27	0.0002	0.15	0.0407	0.16	0.0351	0.20	0.0058
Bilirubin	−0.078	0.2909	−0.18	0.0133	−0.13	0.0711	−0.092	0.2216	−0.11	0.1427
PT	0.021	0.7759	0.12	0.0978	0.066	0.3663	0.088	0.2349	0.14	0.0552
Platelet	0.11	0.1247	0.13	0.080	0.069	0.3444	0.040	0.5908	0.083	0.2585
CONUT	−0.2	0.0051	−0.27	0.0002	−0.16	0.0246	−0.19	0.0088	−0.15	0.0423
AST	−0.11	0.1441	−0.12	0.1023	−0.10	0.1521	−0.094	0.2403	−0.17	0.0181
ALT	−0.03	0.6782	−0.039	0.5944	−0.14	0.0507	−0.087	0.2403	−0.13	0.0662
**Female**	**SF**		**RE**		**MH**		**PCS**		**MCS**	
	***r***	***p* Value**	***r***	***p* Value**	***r***	***p* Value**	***r***	***p* Value**	***r***	***p* Value**
Age	0.017	0.8212	−0.11	0.1373	−0.021	0.7774	−0.20	0.0066	0.097	0.1948
BMI	−0.012	0.8759	−0.0045	0.9511	−0.0075	0.9186	−0.062	0.4035	−0.044	0.5541
HGS	0.18	0.0179	0.19	0.0112	0.17	0.0222	0.28	0.0003	0.15	0.0446
SMI	0.025	0.7319	0.11	0.1375	0.057	0.4395	0.087	0.2433	−0.060	0.4255
Albumin	0.20	0.0072	0.19	0.0098	0.12	0.0878	0.30	<0.0001	0.15	0.0481
Bilirubin	−0.18	0.0148	−0.16	0.0304	−0.04	0.5927	−0.21	0.0043	−0.074	0.3295
PT	0.14	0.0503	0.13	0.0766	0.12	0.1036	0.12	0.1030	0.19	0.0119
Platelet	0.098	0.1837	0.12	0.1067	0.12	0.1103	0.16	0.0311	0.093	0.2115
CONUT	−0.17	0.0253	−0.17	0.0206	0.081	0.2708	−0.31	<0.0001	−0.11	0.1407
AST	−0.16	0.0336	−0.13	0.0719	−0.16	0.0245	−0.11	0.1386	−0.17	0.0206
ALT	−0.084	0.2597	−0.051	0.4811	−0.98	0.1817	−0.025	0.7397	−0.15	0.0462

PF; physical functioning, RP; role physical, BP; bodily pain, GH; general health perception, VT; vitality, SF; social functioning, RE; role emotion, MH; mental health, PCS; physical component summary score, MCS; mental component summary score, BMI; body mass index, HGS; hand grip strength, SMI; skeletal muscle mass index, PT; prothrombin time, CONUT; controlling nutritional score, AST; aspartate aminotransferase, ALT; alanine aminotransferase.

**Table 5:** Multivariate analyses of factors linked to the SF-36 scores in male and female patients.

**Male**	**Parameter (Significant Factor Only)**	**Estimates**	**Standard Error**	***p* Value**
PF	HGS	0.494	0.104	0.0031
	Serum albumin	9.008	3.152	0.0048
	CONUT score	−1.656	0.796	0.0389
RP	HGS	0.579	0.244	0.0185
	Serum albumin	17.593	4.899	0.0004
BP	NA	NA	NA	NA
GH	NA	NA	NA	NA
VT	Serum albumin	8.944	4.158	0.0327
SF	Serum albumin	10.456	3.929	0.0085
RE	Serum albumin	18.765	4.891	0.0002
	ALT	−0.245	0.0845	0.0042
MH	NA	NA	NA	NA
PCS	HGS	0.288	0.141	0.0421
	Serum albumin	11.016	2.692	<0.0001
MCS	NA	NA	NA	NA
**Female**	**Parameter (significant factor only)**	**Estimates**	**Standard error**	***p* Value**
PF	HGS	0.958	0.322	0.0034
RP	NA	NA	NA	NA
BP	NA	NA	NA	NA
GH	NA	NA	NA	NA
VT	HGS	0.851	0.346	0.0150
SF	NA	NA	NA	NA
RE	HGS	0.729	0.36	0.0422
MH	AST	−0.124	0.0609	0.0440
PCS	HGS	0.521	0.22	0.0191
MCS	NA	NA	NA	NA

PF; physical functioning, RP; role physical, BP; bodily pain, GH; general health perception, VT; vitality, SF; social functioning, RE; role emotion, MH; mental health, PCS; physical component summary score, MCS; mental component summary score, HGS; hand grip strength, CONUT score; controlling nutritional score, NA; not applicable, ALT; alanine aminotransferase, AST; aspartate aminotransferase.

## Abbreviation

H-QOL	health-related quality of life
CLD	chronic liver disease
SF-36	36-Item Short Form Health Survey
SMM	skeletal muscle mass
JSH	Japanese Society of Hepatology
HGS	hand grip strength
d-HGS	decreased HGS
nd-HGS	non-decreased HGS
BIA	bioimpedance analysis
BMI	body mass index
SMI	skeletal muscle mass index
d-SMM	decreased SMM
nd-SMM	non-decreased SMM
LC	liver cirrhosis
CONUT score	controlling nutritional score
PF	physical functioning
RP	role physical
BP	bodily pain
GH	general health perception
VT	vitality
SF	social functioning
RE	role emotion
MH	mental health
PCS	physical component summary score
MCS	mental component summary score
SD	standard deviation
CI	confidence interval
